# Integration of miRNA expression analysis of purified leukocytes and whole blood reveals blood-borne candidate biomarkers for lung cancer

**DOI:** 10.1080/15592294.2024.2393948

**Published:** 2024-08-20

**Authors:** Guini Hong, Yue Huo, Yaru Gao, Liyuan Ma, Shuang Li, Tian Tian, Haijian Zhong, Hongdong Li

**Affiliations:** a School of Medical Information Engineering, Gannan Medical University, Ganzhou, China; b School of Public Health and Health Management, Gannan Medical University, Ganzhou, China

**Keywords:** Lung cancer, leukocyte proportion, relative expression ordering, circulating biomarker

## Abstract

Changes in leukocyte populations may confound the disease-associated miRNA signals in the blood of cancer patients. We aimed to develop a method to detect differentially expressed miRNAs from lung cancer whole blood samples that are not influenced by variations in leukocyte proportions. The Ref-miREO method identifies differential miRNAs unaffected by changes in leukocyte populations by comparing the within-sample relative expression orderings (REOs) of miRNAs from healthy leukocyte subtypes and those from lung cancer blood samples. Over 77% of the differential miRNAs observed between lung cancer and healthy blood samples overlapped with those between myeloid-derived and lymphoid-derived leukocytes, suggesting the potential impact of changes in leukocyte populations on miRNA profile. Ref-miREO identified 16 differential miRNAs that target 19 lung adenocarcinoma-related genes previously linked to leukocytes. These miRNAs showed enrichment in cancer-related pathways and demonstrated high potential as diagnostic biomarkers, with the LASSO regression models effectively distinguishing between healthy and lung cancer blood or serum samples (all AUC > 0.85). Additionally, 12 of these miRNAs exhibited significant prognostic correlations. The Ref-miREO method offers valuable candidates for circulating biomarker detection in cancer that are not affected by changes in leukocyte populations.

## Background

Blood samples have become increasingly popular for cancer biomarker research due to their convenience, low cost, and minimally invasive nature [1–3]. However, analyzing blood samples presents challenges because they contain various types of leukocytes. Diseases such as cancer and inflammatory conditions often affect leukocyte subtype populations, leading to changes in their proportions in the blood [4,5]. These alterations can introduce noise or bias into disease association studies, complicating the differentiation between genuine disease-related changes and those resulting from variations in cell composition. To overcome this, researchers have developed algorithms, categorized as reference-free or reference-based, to separate cell types or estimate their proportions [6–9]. However, biomarkers from reference-free methods must account for disease-related cell proportion effects. Meanwhile, reference-based algorithms can be challenging in retaining their performance in independent groups as they have yet to be trained on a diverse study population [9]. Additionally, these algorithms are sensitive to batch effects and genetic variations. Therefore, there is a need to develop more robust, accurate, and adaptable algorithms to deal with the problems posed by changes in leukocyte proportions in blood samples, especially in studying cancer biomarkers.

MicroRNAs (miRNAs), a class of small non-coding RNA molecules, play a regulatory role in post-transcriptional gene expression [10]. Numerous studies have developed miRNA biomarkers for disease diagnosis using genome-wide blood miRNA expression profiles [11]. However, differentially expressed miRNAs in disease blood could also be influenced by the composition of leukocyte proportions [12]. Further research on developing methods to estimate and correct cell proportions at the molecular level of miRNAs are warranted. Although methods commonly used for immune cell infiltration analysis, such as CIBERSORT [13], could theoretically estimate the proportions of different leukocyte types based on miRNA expression profiles, they require cell type-specific miRNA expression features currently unavailable for analysis. Therefore, it remains unclear to what extent disease-associated miRNA signals in cancer blood are affected by cell composition shifts, making it challenging to identify disease-associated genetic miRNA changes as diagnostic biomarkers.

Within-sample relative expression orderings (REOs) or relative methylation orderings (RMOs) of genes have been reported to be insensitive to systematic biases, invariant to monotonic data normalization, and robust against inter-individual genetic differences at the molecular level [14]. We have developed two methods to identify genuine disease-associated expression or methylation alterations from mixed-cell blood samples based on consistent REO or RMO patterns predefined in healthy purified leukocyte subtypes [15,16]. These methods effectively detect disease-associated differential signals originating from changes in intracellular molecules within leukocytes in whole blood samples and have been applied in Alzheimer’s disease [17], ovarian cancer [16], and other diseases [15]. Therefore, in this study, we will utilize REOs of miRNAs to develop a method to identify disease-related alterations from mixed-cell whole blood.

Our study has two main objectives. Our primary objective was to assess the influence of alterations in leukocyte proportions on the differential miRNA signals detected in lung cancer patients compared to healthy controls. To achieve this, we identified subtype-specific miRNA expression profiles in purified leukocyte subtypes by clustering analysis and detected miRNA features unique to each subtype. Using the single-sample gene set enrichment analysis (ssGSEA) algorithm, we demonstrated changes in leukocyte proportions under lung cancer conditions. Then, we assessed whether these changes explained the differential miRNAs observed between cancerous and healthy blood samples. We also aimed to develop a method for identifying cellular differential miRNA signals in whole blood samples from lung cancer patients based on within-sample REOs of miRNAs. We finally evaluated the diagnostic and prognostic potential of the differential miRNA signals identified by the developed Ref-miREO algorithm to demonstrate its practicality and effectiveness.

## Material and methods

### Data source and preprocessing

The miRNA expression profile data analyzed in this study were obtained from the GEO and ArrayExpress databases (Table 1). The datasets GSE28487 and GSE28489 assayed the miRNA expression of leukocytes in various subtypes in healthy individuals [18]. GSE28487 assayed nine leukocyte subtypes, while GSE28489 assayed seven leukocyte subtypes. These two datasets were utilized as the training set. In contrast, the datasets GSE98830 [19] and GSE55993 [20] had six and five healthy leukocyte subtypes, respectively, and were employed as independent validation sets.

**Table 1.** Datasets analyzed in this study.

Data	Origin	Sample No.	Platform
GSE28487	Purified leucocytes	Monocytes: 9, Neutrophils: 4, Eosinophils: 4, B cells: 5, CD4+ T cells: 4, CD8+ T cells: 5, NK cells: 5, mDCs: 2, pDCs: 5	Affymetrix Multispecies miRNA-1 Array
GSE28489	Purified leucocytes	Monocytes: 5, Neutrophils: 5, Eosinophils: 3, B cells: 5, CD4+ T cells: 5, CD8+ T cells: 5, NK cells: 5	Affymetrix Multispecies miRNA-1 Array
GSE98830	Purified leucocytes	Monocytes: 6, Granulocytes: 5, B-cells: 5, CD4+ T cells: 5, CD8+ T cells: 5, NK cells: 5	Agilent -021,827 Human miRNA Microarray (V3)
GSE55993	Purified leucocytes	Monocytes: 6, Neutrophils: 7, T cells: 6, B cells: 6, NK cells: 6	Agilent -031,181 Unrestricted_Human_miRNA_V16.0_Microarray
E-MTAB-8026	Whole blood	Healthy: 964, Lung Cancer: 606, Non-cancer pulmonary diseases: 593	Agilent -070,156 Human miRNA
GSE17681	Whole blood	Lung cancer: 17, Healthy controls: 19	febit Homo Sapiens miRBase 13.0
GSE137140	Serum	Lung cancer: 1566, Non-cancer controls: 2178	3D-Gene Human miRNA V21_1.0.0
GSE198958	Exosome	Non-small cell lung cancer: 67	Agilent -070,156 Human miRNA_V21.0 Microarray

The dataset E-MTAB-8026 consists of miRNA expression data from whole blood samples of 964 healthy individuals, 606 cases of lung cancer, and 593 cases of non-tumor lung disease (NTLD) [21]. Four hundred randomly selected samples from each group were used for training, while the rest were used for testing. GSE17681 includes miRNA expression profiles from 17 lung cancer and 19 healthy blood samples [22]. GSE137140 contains serum microRNA profiles from 3,924 samples, including 1,566 preoperative lung cancer, 180 postoperative lung cancer, and 2,178 non-cancer controls [23]. Only the preoperative lung cancer and non-cancer samples were analyzed in the study. These two datasets were used to validate the performance of the miRNA diagnostic model.

GSE198958 provided information on exosome miRNA expression and survival of 67 non-small cell lung cancer patients [24], which was used to assess the prognostic capability of the identified differential miRNAs.

Due to the inconsistencies in the platforms used for each dataset, miRNA names were cross-referenced with miRBase V21 using the miRNA Enrichment Analysis and Annotation Tool (miEAA) [25], which converted them into a standardized miRNA nomenclature. After preprocessing, 777 miRNAs detected in all datasets were used for subsequent analyses. Since all miRNAs analyzed in this study are of human origin, the ‘hsa-’ prefix was omitted from their names.

### Identification of leukocyte-specific miRNAs

The miRNAs were considered to be specifically expressed in a particular leukocyte subtype when they showed differential upregulation compared to other cell types. Limma was used for the differential analysis [26].

### Evaluation of proportion changes of each leukocyte subtype in whole blood

Utilizing the specific list of miRNAs identified for each leukocyte, we employed the ssGSEA algorithm to calculate the enrichment scores of different leukocyte subtypes. By utilizing these scores, we can determine the relative changes in the proportions of different leukocytes in whole blood.

### Application of reference-free and reference-based methods to whole blood miRNA profiles

We analyzed two methods developed to mitigate cell-composition effects of whole blood: reference-based and reference-free. The reference-based method operates via deconvolution to estimate the proportion of each leukocyte subtype in blood and adjust them. Using leukocyte-specific miRNAs as a reference, we estimated the absolute proportions of each type of leukocyte in whole blood samples using the algorithm provided by Houseman et al [8]. The reference-free approach via surrogate variable analysis (SVA) is used to identify and adjust potential confounding factors [7]. We applied SVA to miRNA expression data using the *SVA* package in R with default parameter settings.

### Identification of differential miRNAs derived from leukocytes in disease whole blood samples

**Figure 1.**
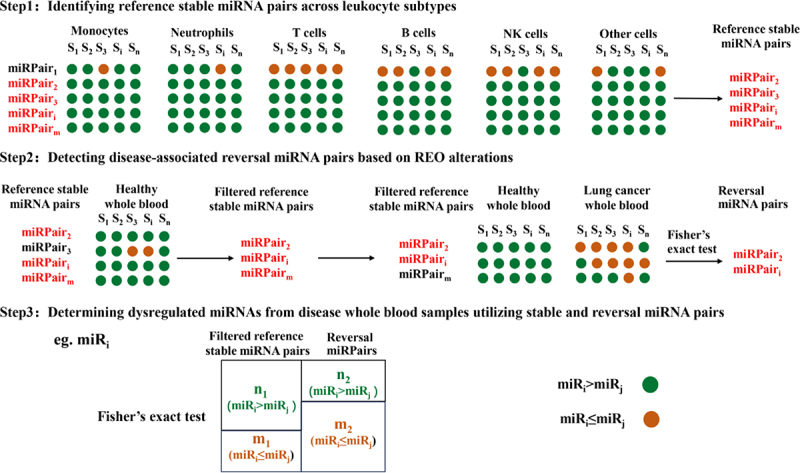
Overall workflow for the Ref-miREO method.

A workflow for identifying differentially expressed miRNAs derived from leukocytes in lung cancer blood samples was outlined based on the reference of the REO patterns of miRNAs in subtypes of leukocytes (Figure 1).

(1) Identifying reference stable miRNA pairs across leukocyte subtypes

A miRNA pair (miR_i_, miR_j_) is deemed stable if miR_i_ consistently exhibits higher expression levels than miR_j_ across all leukocyte subtypes within a dataset. The REO of such a stable miRNA pair could remain consistent across all subtypes, irrespective of changes in the proportions of leukocytes, unless there are alterations in the miRNA expression levels within the individual subtypes. Those miRNA pairs commonly determined as stable in GSE28487 and GSE28489 are referred to as reference stable miRNA pairs.

(2) Detecting disease-associated reversal miRNA pairs based on REO alterations

We detected significantly reversed miRNA pairs by evaluating changes in the REOs of stable miRNA pairs in disease samples, utilizing the training set of the E-MTAB-8026 dataset. First, healthy blood samples from the training set were used to filter for reference stable gene pairs with consistent REOs. For a filtered reference stable miRNA pair, if the REO pattern miR_i_>miR_j_ presents in *n*_1_ healthy samples and *n*_2_ disease samples, and miR_i_≤miR_j_ presents in *m*_1_ healthy samples and *m*_2_ disease samples, respectively, we can calculate the significance level (*p*-value) of the distribution difference in the REOs of the miRNA pair using Fisher’s exact test and adjust the *p*-value through a false discovery rate (FDR) correction [27]. If a stable miRNA pair meets the criterion of *P*_healthy_(miR_i_>miR_j_)≥0.9, ∆*P*=*P*_healthy_(miR_*i*_>miR_*j*_)- *P*_disease_(miR_i_>miR_*j*_)≥0.15, and FDR ≤ 0.05, it is identified as a reversal miRNA pair. Here, *P*_healthy_(miR_*i*_>miR_*j*_)≈*n*_1_/(*n*_1_+*m*_1_), and *P*_disease_(miR_*i*_> miR_*j*_)≈*n*_2_/(*n*_2_+*m*_2_).

(3) Determining dysregulated miRNAs from disease whole blood samples utilizing stable and reversal miRNA pairs

Using the stable miRNA pairs that meet the criteria *P*_healthy_(miR_i_>miR_j_)≥0.9 as a reference and the reversal miRNA pairs identified in Step 2, the process for identifying differentially expressed miRNAs is outlined as follows. First, for each miRNA (miR_*i*_) involved in the reversal pairs, calculate the number of samples (*n*_1_, *m*_1_, *n*_2_, and *m*_2_) exhibiting miR_*i*_>miR_*j*_ and miR_*j*_≤miR_*i*_in both stable and reversal pairs, respectively. Fisher’s exact test is then utilized to determine the significance level (*p*-value) of the distribution difference between healthy and disease samples. Subsequently, the miRNA with the smallest *p*-value is chosen as a differentially expressed miRNA. If the ratio *n*_1_/*m*_1_ exceeds *n*_2_/*m*_2_, it suggests the miRNA’s expression is downregulated in disease samples; conversely, it suggests upregulation. The selected differentially expressed miRNA and its corresponding miRNA pairs are then eliminated from the reversal pairs. This process is repeated until no additional differentially expressed miRNAs can be identified. All chosen miRNAs are considered differentially expressed miRNAs and will be used for further analyses.

### Evaluation of the diagnostic and prognostic potential of differential miRNAs

To assess the predictive performance of the differentially expressed miRNAs detected by Ref-miREO in lung cancer blood samples, a 10-fold Lasso regression was performed on the training set. The model with the lowest penalized likelihood deviance (λ value) was chosen as the predictive model. The performance was evaluated using the area under the curve (AUC) of the ROC. The diagnostic efficacy of the model was further evaluated in the testing group and the two additional independent validation sets. Cox regression analysis was employed to examine the correlation between the differential expression of miRNAs and the prognosis of the disease.

### Functional enrichment analysis and target prediction of differential miRNAs

Functional enrichment analysis of the differentially expressed miRNAs was conducted utilizing the miEAA online tool. The miRNA target genes were identified by querying the miRNet database [28], which provides literature-supported miRNA-target gene interactions.

### Supplementary material

Supplementary Table S1 lists highly expressed miRNAs specific to each leukocyte subtype. Supplementary Figure S1 shows the boxplot of the six leukocyte subtypes in normal whole blood samples estimated by deconvolution method.

## Results

### Leukocyte subtype-specific miRNA expression and proportion changes under disease condition

**Figure 2.**
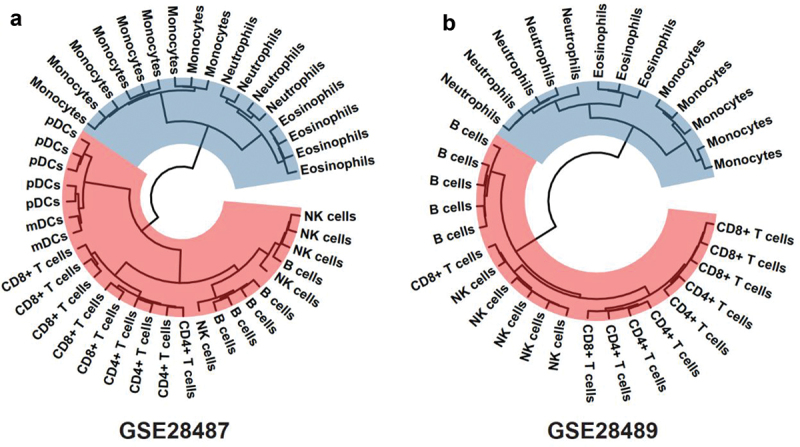
Hierarchical clustering of miRNA expression in purified leukocyte subtypes in GSE28487 (a) and GSE28489 (b).

To explore the expression of miRNAs among various leukocyte subtypes, we performed a hierarchical clustering analysis based on the two datasets of purified leukocyte subtypes (GSE28487 and GSE28489). As shown in Figure 2, the leukocyte subtypes exhibited clear clustering patterns, with leukocytes of the same subtype clustering closely together. Interestingly, monocytes, neutrophils, DCs, and eosinophils clustered together. They belonged to myeloid-derived cells. At the same time, the lymphoid-derived T cells, B cells, and NK cells were in a separate group. The results suggested that leukocytes show subtype-specific miRNA expression profiles.

Then, we evaluated potential alterations in the leukocyte populations under disease conditions. First, we identified highly expressed miRNAs exclusive to monocytes, neutrophils, eosinophils, B cells, T cells, and NK cells using the GSE28487 dataset, which encompasses a larger sample size. A miRNA was deemed specific to a particular leukocyte subtype if it exhibited significant upregulation in that subtype compared to other leukocyte types (see Methods). The miRNAs specific to each leukocyte subtype are detailed in Supplementary Table S1.

**Figure 3.**
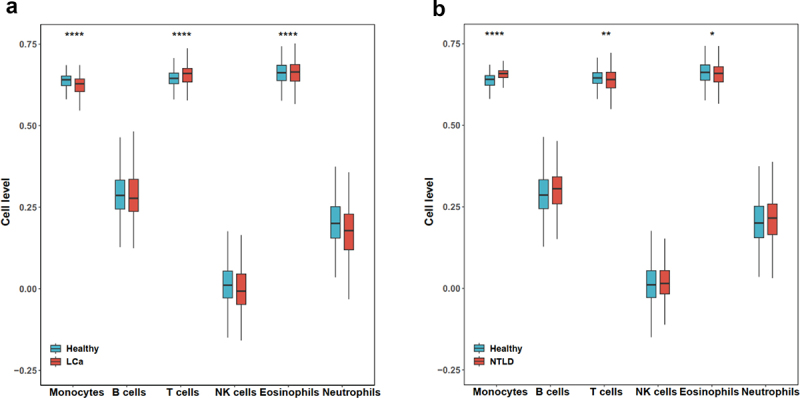
Boxplot of changes in leukocyte composition. (a) feature miRNA scores in lung cancer and control PWB samples; (b) feature miRNA scores in non-cancer lung disease and control PWB samples. The legend is as following: *p<0.05; **p<0.01; ****p<0.0001 (Student’s *t*-test). Abbreviations: LCa: lung cancer; NTLD: non-tumor lung disease.

Using the ssGSEA algorithm, we calculated scores for each feature miRNA set for leukocytes in the training set based on the highly expressed miRNAs specific to each leukocyte subtype. The results showed that in the whole blood, the feature miRNA scores for monocytes and neutrophils were significantly lower in lung cancer samples compared to healthy samples (Figure 3a). On the other hand, T cell feature miRNA scores were significantly higher in lung cancer. In contrast, the monocyte feature miRNA scores were significantly higher in NTLD samples than in healthy samples, while the T cell feature miRNA scores were significantly lower (Figure 3b).

These results indicate that the populations of leukocyte subtypes may undergo alterations in the context of disease. As the miRNA expression observed in whole blood samples can be considered as a composite of leukocyte-specific miRNA levels weighted by their respective proportions, these alterations in proportions have the potential to introduce additional signals in miRNA expression analysis of whole blood samples, given the subtype-specific expression profiles of miRNAs in leukocytes.

### Influence of leukocyte proportion changes on disease-associated differential miRNA signals in whole blood

**Figure 4.**
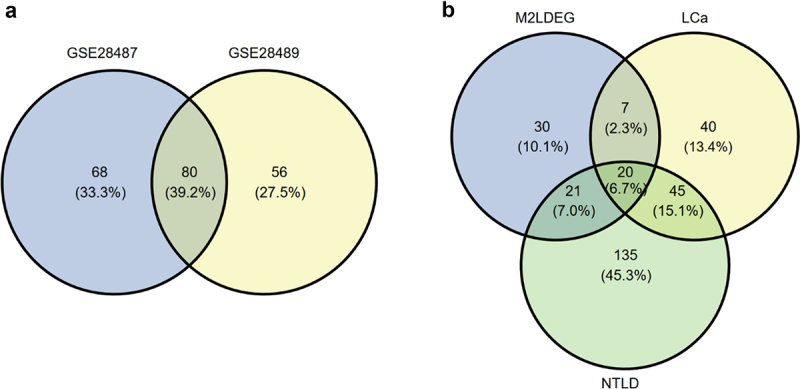
Venn diagrams of differential miRNAs. (a) overlaps of differential miRNAs in purified leukocyte subtypes from GSE28487 and GSE28489. (b) overlaps of differential miRNAs identified from purified leukocyte subtypes, lung cancer blood samples, and non-tumor lung disease blood samples. Limma was used for the differential analysis. Abbreviations: M2LDEG: differentially expressed miRNAs in myeloid-derived cells compared to lymphoid-derived cells; LCa: lung cancer; NTLD: non-tumor lung disease.

In previous studies, we have confirmed that comparing differential myeloid to lymphoid genes with the differential genes identified between disease and healthy blood samples helps determine if changes in leukocyte proportion affect the differential signals identified in the whole blood of disease [5,29]. Therefore, we first detected differentially expressed miRNAs between the two clusters, myeloid-derived cells and lymphoid-derived cells. With the criteria |log2FC|≥0.26 (1.2-fold change) and FDR ≤ 0.05, we identified 148 and 136 miRNAs as differentially expressed in GSE28487 and GSE28489, respectively. Among them, 80 miRNAs were identified in both datasets (*p* = 7.88371 × 10^−32^, cumulative hypergeometric test, Figure 4a). Notably, 78 out of these 80 miRNAs showed consistent upregulation or downregulation patterns in myeloid-derived cells relative to lymphoid-derived cells across both datasets.

Then, we analyzed the differential miRNAs in the whole blood of lung cancer patients. With the criteria |log2FC|≥0.26 (1.2-fold change) and FDR ≤ 0.05, we identified 112 differential miRNAs between lung cancer and healthy samples. Among them, 27 miRNAs overlapped with the 78 consistently differential miRNAs in leukocytes (*p* = 1.54 × 10^−6^, cumulative hypergeometric test, Figure 4b), and 21 (77.77%) of the overlaps exhibited opposite changes in lung cancer compared to healthy samples as seen in the myeloid-derived cells relative to lymphoid-derived cells. At the same criteria, we detected 221 differential miRNAs for NTLD. Forty-one miRNAs overlapped with the 78 consistently differential miRNAs (*p* = 1.84 × 10^−16^, cumulative hypergeometric test, Figure 4b), and 33 (80.49%) of the overlapped miRNAs showed consistent changes in NTLD compared to healthy samples, as seen in the myeloid-derived cells relative to lymphoid-derived cells.

These results suggest that disease-associated differential miRNAs detected in whole blood samples reflect shifts in leukocyte populations to some extent.

### Reference-based deconvolution and surrogate variable analysis applied to the training miRNA data

To identify the leukocyte-derived miRNA alterations associated with lung cancer, we evaluated the published reference-based deconvolution and reference-free SVA methods, which have been designed to mitigate the cell-composition effects of whole blood.

Using the mean expression of specific miRNAs for each leukocyte subtype in GSE28487 and GSE28489 (Supplementary Table S1), respectively, as a reference, the deconvolution algorithm estimated cell proportions in the training set. The results showed significant differences in estimated cell proportions when using different references (Supplementary Figure S1). For example, the proportions of B cells were 0.019 ± 0.05 and 0.329 ± 0.07, respectively, when using GSE28487 and GSE28489 as reference. Meanwhile, the average proportions of monocytes were estimated to be higher than 0.566, significantly higher than the actual proportion ranging from 0.03 to 0.1. Due to the incorrect estimation of absolute proportions, we did not pursue the identification of differential miRNAs using the reference-based deconvolution method.

We identified one primary surrogate variable from the training set using the SVA algorithm and detected 112 differential miRNAs after adjusting the surrogate variable with FDR < 0.05 and |logFC|>0.26. However, we found these miRNAs consistent with the 112 differential miRNAs using the Limma tool. This analysis suggested that SVA could not efficiently eliminate the impact of such leukocyte proportion changes.

### Identification of altered miRNAs in whole blood of lung cancer based on stable REOs between purified leukocytes

To identify differential signals indicative of genetic alterations within leukocytes in whole blood samples from lung cancer, we developed Ref-miREO. We identified 51,672 and 79,171 miRNA pairs whose REOs were stable in all leukocyte subtypes in the two training leukocyte datasets, respectively, with 33,218 overlapping miRNA pairs between them (*p* < 2.2 × 10^−16^, cumulative hypergeometric test). Among these 24,453 miRNA pairs remained stable in 90% of healthy whole blood samples in the training set of lung cancer (*p* = 4.11 × 10^−195^, cumulative hypergeometric test). Using these 24,453 miRNA pairs as a reference, we identified 115 miRNA pairs with reversal REOs in lung cancer whole blood samples under the criteria ΔP > 0.15 and FDR ≤ 0.05 (Fisher’s exact test, see Methods). Although the number of reversal miRNA pairs was small, no significant reversal miRNA pairs were found in 100 random permutation experiments of the training set by disturbing the sample labels. Based on the 24,453 stable pairs and 115 reversal pairs, we identified 16 differentially expressed miRNAs (Table 2).

**Table 2.** Differential miRNAs detected from lung cancer whole blood samples by Ref-miREO.

miRNA	dysregulated	*q*-value^†^	HR (95%CI)^‡^	*p*-value^§^
miR-200b-3p	−1	7.23×10^−19^	2048(29.45-1.42×10^3^)	4.27×10^−4^
miR-17-5p	−1	7.05×10^−11^	1.33(1.06-1.65)	1.18×10^−2^
miR-20a-5p	−1	2.45×10^−6^	1.26(1.03-1.54)	2.46×10^−2^
miR-18a-5p	−1	1.75×10^−6^	1.59(1.11-2.28)	1.14×10^−2^
miR-29b-2-5p	−1	4.41×10^−4^	6.02(0.009-4087)	0.59
miR-93-5p	−1	8.36×10^−4^	1.44(1.10-1.90)	7.99×10^−3^
miR-186-5p	1	3.23×10^−3^	1.95(1.19-2.69)	7.97×10^−3^
miR-30d-5p	1	8.51×10^−3^	1.37(1.10-1.70)	4.24×10^−3^
miR-320d	1	1.08×10^−2^	1.78(1.19-2.69)	5.21×10^−3^
miR-324-3p	1	1.52×10^−2^	1.77(0.84-3.76)	0.13
miR-575	1	1.51×10^−2^	1.41(0.93-2.13)	0.10
miR-16-5p	−1	1.68×10^−2^	1.15(0.94-1.43)	0.17
miR-151a-3p	1	1.64×10^−2^	1.49(1.13-1.98)	4.60×10^−3^
miR-484	1	1.77×10^−2^	1.77(1.24-2.50)	1.31×10^−2^
miR-191-5p	−1	4.03×10^−2^	13.38(1.64-109.29)	1.55×10^−2^
miR-20b-5p	−1	3.99×10^−2^	1.33(1.03-1.71)	2.68×10^−2^

^**†**^Fisher’s exact test *p*-value adjusted by BH algorithm; ^**‡**^Cox regression analysis Hazard ratio (HR) and 95% confidence interval (CI); ^§^Cox regression analysis Wald test *p*-value.

### Target genes of detected differential miRNAs by ref-miREO

**Figure 5.**
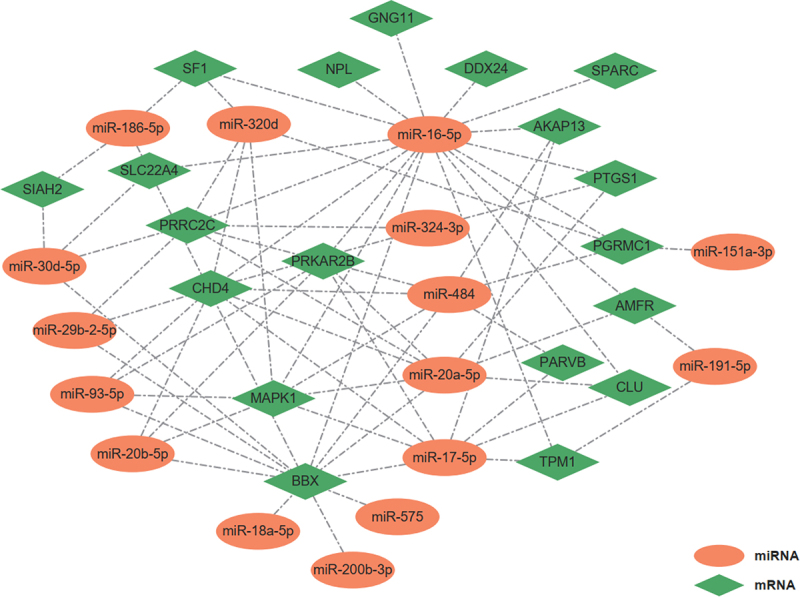
The mRNA-miRNA regulation network.

Our previous study has identified 22 differential genes derived from leukocytes in the whole blood of lung adenocarcinoma patients [15]. Using the miRNet database, we discovered that 937 miRNAs were implicated in the regulation of these 22 genes. Of these miRNAs, 456 were among the 777 miRNAs analyzed in our study. Importantly, the 16 differential miRNAs identified by Ref-miREO exhibited significant regulatory relationships with 19 of the 22 leukocyte-derived genes (*p* = 1.77 × 10^−4^, cumulative hypergeometric test), forming a regulatory network comprising 72 edges (Figure 5). This result indicated that the differential miRNAs detected by Ref-miREO likely reflect genuine miRNA changes within leukocytes rather than changes in leukocyte proportions.

**Figure 6.**
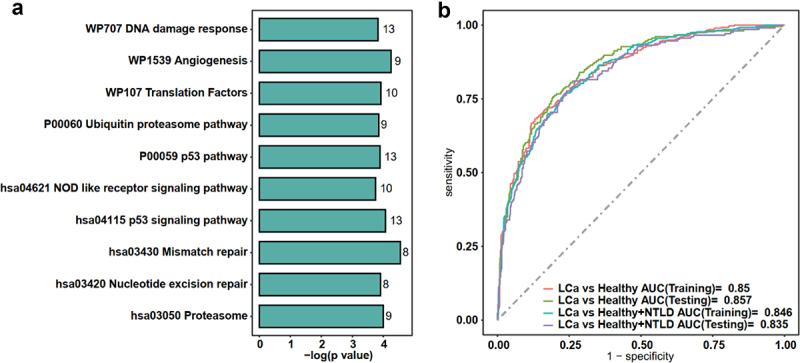
(a) the top 10 pathways enriched for the differential miRNAs detected from whole blood samples of lung cancer by ref-miREO; (b) the AUC for predicting lung cancer and non-tumor lung disease. Abbreviations: LCa: lung cancer; NTLD: non-tumor lung disease.

Functional enrichment analysis of these 16 miRNAs was conducted through miEAA. The results showed that the target genes of these miRNAs were mainly enriched in cancer-related pathways such as the p53 signaling pathway, DNA damage response, and proteasome, as shown in the top 10 pathways in Figure 6a.

### Prediction performance and prognostic evaluation of differential miRNAs detected by ref-miREO

To assess the diagnostic efficacy of the differential miRNAs detected by Ref-miREO, we used Lasso regression to construct predictive models for distinguishing lung cancer patients from healthy individuals. The results revealed that the constructed regression models using these 16 miRNAs exhibited a significant ability to differentiate lung cancer samples. Specifically, in both the training and test sets, the AUC values for the constructed model were 0.850 and 0.857 (Figure 6b). In the validation whole blood dataset for lung cancer (GSE17681), the AUC value for the constructed model was 1. Notably, in the validation serum dataset (GSE137140), the AUC value for the constructed model reached 0.9591, indicating the possibility that altered miRNAs observed in serum miRNAs can derive from leukocytes.

In the training set, we combined healthy samples with NTLD samples to create a model to distinguish from lung cancer patients. The results showed AUC values of 0.846 and 0.835 in the training and test sets, respectively, suggesting that the differential miRNAs could differentiate between non-cancer controls and lung cancer samples.

The GSE198958 dataset contains the expression profiles of miRNAs in extracellular vesicles from 67 non-small cell lung cancer patients and includes their survival information. Univariate Cox regression analysis revealed that 12 of the 16 miRNAs were significantly associated with the patient’s prognosis (*p* = 1.56 × 10^−4^, Wald test).

This result indicates that the differentially expressed miRNAs identified in the whole blood of lung cancer by Ref-REO hold promising potential as biomarkers for lung cancer diagnosis and prognosis.

## Discussion

In this study, we focused on identifying diagnostic biomarkers for cancer using blood samples, considering the heterogeneous nature of blood, which consists of various leukocytes. Previous studies have already explored the issue of detecting disease-associated changes at the cellular level in mixed-cell blood samples, suggesting that alterations in leukocyte proportions may mask genetic and epigenetic signals associated with disease [5,29]. In this paper, we extended these analyses to the miRNA molecular level, hypothesizing that identifying miRNA-based biomarkers for disease diagnosis using blood samples also faces additional challenges due to the influence of leukocyte proportions on abnormal miRNA signals.

To tackle this challenge, we first demonstrated that different types of leukocytes exhibited different patterns of miRNA expression. Then, we used the ssGSEA algorithm to determine how the proportions of leukocytes changed. We found a decrease in monocytes and neutrophils from the myeloid lineage but an increase in T cells from the lymphoid lineage in lung cancer compared to healthy blood samples. Additionally, when we compared the differential miRNAs between the myeloid and lymphoid lineage leukocytes to the differential signals directly detected between disease and healthy blood samples, we found a significant overlap, illustrating that changes in miRNA levels in whole blood are related to the underlying leukocyte population changes. Therefore, it is essential to identify changes in miRNAs from mixed-cell blood samples unaffected by leukocyte population changes under disease conditions.

Our previous studies have introduced the concept of REOs or RMOs derived from microarray or sequencing measurements, which could not be affected by systematic biases, data normalization, or inter-individual genetic differences. Based on the within-sample REOs of miRNAs defined in this study, we developed the Ref-miREO method to identify differential miRNA signals that could reflect intrinsic genetic changes of leukocytes in whole blood samples from lung cancer patients. We also assessed the diagnostic and prognostic potential of the differential miRNA signals identified by the Ref-miREO algorithm, demonstrating its practicality and effectiveness.

In our study, we utilized two independent datasets, one from whole blood and the other from serum, to further examine the Ref-miREO method. Both datasets demonstrated that the differential miRNAs exhibited high discriminatory power between lung cancer and healthy individuals, evidenced by an AUC > 0.95. To further enhance the reliability of our findings, we plan to employ real-time PCR techniques to validate these differential miRNAs in a prospective cohort of lung cancer patients in the future. This additional validation step will strengthen the robustness of our results and provide more confidence in the potential diagnostic biomarkers identified for lung cancer.

Ref-miREO is a reference-dependent method that relies on stable miRNA pairs identified from various leukocyte subtypes. However, different experimental conditions and biological factors can influence the number of stable pairs. To discuss this problem, we undertook a sensitivity analysis to assess whether the differential miRNAs could be consistently identified by using independent datasets of purified leukocyte subtypes. In the GSE55993 dataset, we found 152,464 stable miRNA pairs across all leukocyte subtypes, and in GSE98830, we identified 65,512 stable miRNA pairs. These two lists of stable pairs significantly intersected with those from the trained leukocyte subtypes (both *p* < 0.05, cumulative hypergeometric test). We detected 60 and 32 differentially expressed miRNAs using these stable miRNA pairs in the respective datasets. Remarkably, 13 of the 16 differential miRNAs identified using the trained leukocyte subtypes overlapped with the 60 miRNAs, and 10 of the 16 differential miRNAs overlapped with the 32 miRNAs. This sensitivity analysis demonstrates the robust performance of the algorithm in consistently detecting differential miRNAs across different datasets of purified leukocyte subtypes.

In studying lung cancer pathogenesis, we found that relevant miRNAs in the 16 differentials identified by Ref-miREO play significant roles either as oncogenes promoting cancer or as tumor suppressors inhibiting cancer. Notably, miR-200b-3p enhances cancer cell proliferation and metastasis [30]. MiR-575 and miR-484 are also oncogenes that contribute to non-small cell lung cancer progression [31,32]. Conversely, miR-16-5p, a tumor suppressor, is down-regulated, increasing cancer signal activity [33]. Similarly, miR-93-5p and miR-20b-5p are identified as tumor suppressors [34,35]. Additionally, certain miRNAs interact with specific genes, affecting lung cancer’s gene expression, pathogenesis, and progression. For example, miR-17-5p possibly inhibits PLSCR4 gene expression [36], while miR-20a-5p down-regulates HOXB13 expression, inhibiting cell proliferation [37]. MiR-186-5p, on the other hand, targets PTEN, a tumor suppressor, promoting cell growth, migration, and invasion of lung adenocarcinoma [38]. Also, miR-320d and miR-151a-3p are highlighted for their roles in immunotherapy efficacy and neoplasm development [39–41]. Changes in miRNA expression are key in lung cancer, where miR-17-5p and miR-186-5p are up-regulated in non-small cell lung cancer, with the latter also involved in cisplatin resistance and can serve as a diagnostic biomarker [38,42,43]. MiR-20a-5p is linked to decreased disease-free survival in lung squamous cell carcinoma [44], while miR-18a-5p and miR-30d-5p show the potential to affect prognosis and tumor development [45,46].

Nevertheless, it is still not known from which type of leukocyte(s) the disease-associated miRNA alterations detected in whole blood originate. This requires further miRNA expression profiling of purified leukocyte subtypes in disease states to answer this question. In future research, we aim to analyze the leukocyte origin of these disease-specific differential signals observed in whole blood samples.

Our study addresses how leukocyte proportion shifts impact identifying disease-specific differential miRNA signals originating from leukocytes in mixed-cell blood samples. By developing and applying the Ref-miREO method, we provide a potential solution to the limitations of existing methods for blood miRNA biomarker identification in cancer. Our findings contribute to using blood as a noninvasive material for disease diagnosis and prognosis. Future research should aim to validate and refine the Ref-miREO method while extending its application to other types of cancer or diseases.

## Abbreviations

REO: relative expression ordering
RMO: relative methylation ordering
miRNA: microRNA
ssGSEA: single-sample gene set enrichment analysis
mDC: myeloid dendritic cell
pDC: plasmacytoid dendritic cell
NTLD: non-tumor lung diseases
miEAA: miRNA Enrichment Analysis and Annotation
AUC: area under the curve
SVA: surrogate variable analysis

## Supplementary Material

Supplemental Material

Figure S1_CellProp boxplot.pdf

## Data Availability

The datasets analyzed during the current study are available in GEO (http://www.ncbi.nlm.nih.gov/geo/), accession numbers GSE28487, GSE28489, GSE98830, GSE55993, GSE17681, GSE137140 and GSE198958, and in Arrayexpress (https://www.ebi.ac.uk/arrayexpress) with accession number E-MTAB-8026.
