# Supplementary material for: Integration of miRNA expression analysis of purified leukocytes and whole blood reveals blood-borne candidate biomarkers for lung cancer
Source: Epigenetics. 2024 Aug 20;19(1):2393948. doi: 10.1080/15592294.2024.2393948 (PMC11340745; doi:10.1080/15592294.2024.2393948)
Supplement: Supplemental Material [file KEPI_A_2393948_SM8478.doc]

Table S1 MiRNAs specific for each leukocyte subtype

| **subtype** | **miRNA^†^** |
| --- | --- |
| **GSE28487** | |
| **Monocyte** | miR-103a-3p; miR-107; miR-1307-3p; miR-15a-3p; miR-16-5p; miR-17-3p; miR-17-5p; miR-181a-3p; miR-188-5p; miR-18a-3p; miR-18a-5p; miR-18b-5p; miR-20a-5p; miR-21-3p; miR-21-5p; miR-27a-3p; miR-27a-5p; miR-324-5p; miR-345-5p; miR-362-5p; miR-378a-3p; miR-378a-5p; miR-422a; miR-423-3p; miR-500a-3p; miR-501-3p; miR-502-3p; miR-532-3p; miR-532-5p; miR-574-3p; miR-660-5p; miR-708-5p |
| **B cell** | miR-1228-3p; miR-138-5p; miR-1825; miR-195-5p; miR-520g-3p |
| **T cell** | miR-1275; miR-146a-5p; miR-146b-3p; miR-150-3p; miR-150-5p; miR-31-5p; miR-320b; miR-320d; miR-342-3p; miR-342-5p; miR-874-3p; miR-92a-3p |
| **NK cell** | miR-1228-5p; miR-1268a; miR-129-5p; miR-149-3p; miR-346; miR-602; miR-675-5p; miR-758-3p; miR-885-5p |
| **Eosinophil** | miR-100-5p; miR-1246; miR-130b-3p; miR-221-3p; miR-425-5p; miR-652-3p; miR-935 |
| **Neutrophil** | miR-143-3p; miR-145-5p; miR-182-5p; miR-199a-3p; miR-338-5p; miR-551a; miR-593-3p |
| **GSE28489** | |
| **Monocyte** | miR-103a-3p; miR-107; miR-1205; miR-1301-3p; miR-1307-3p; miR-130a-5p; miR-17-5p; miR-181a-3p; miR-188-5p; miR-18a-5p; miR-18b-5p; miR-21-3p; miR-324-3p; miR-337-3p; miR-342-5p; miR-361-3p; miR-362-3p; miR-377-5p; miR-378a-3p; miR-412-3p; miR-421; miR-422a; miR-484; miR-500a-3p; miR-501-3p; miR-502-3p; miR-532-3p; miR-532-5p; miR-574-3p; miR-660-5p; miR-671-3p; miR-671-5p; miR-98-5p |
| **B cell** | miR-138-5p; miR-155-5p; miR-195-5p; miR-222-5p; miR-29b-3p; miR-29c-5p; miR-486-3p; miR-663b |
| **T cell** | miR-146a-5p; miR-150-5p; miR-31-5p; miR-340-5p; miR-342-3p |
| **NK cell** | miR-1181; miR-1228-3p; miR-152-3p; miR-181a-2-3p; miR-181a-5p; miR-181b-5p; miR-181c-5p; miR-20b-5p; miR-28-3p; miR-28-5p; miR-363-3p; miR-638; miR-675-5p |
| **Eosinophil** | miR-100-5p; miR-1246; miR-921; miR-935 |
| **Neutrophil** | miR-125a-5p; miR-143-5p; miR-145-5p; miR-193a-5p; miR-200b-3p; miR-202-3p; miR-223-5p; miR-338-3p; miR-491-5p; miR-628-5p; miR-629-5p; miR-941 |

**^†^**human miRNA prefix () was omitted.
