# Supplementary figures and images for: Integration of miRNA expression analysis of purified leukocytes and whole blood reveals blood-borne candidate biomarkers for lung cancer
Source: Epigenetics. 2024 Aug 20;19(1):2393948. doi: 10.1080/15592294.2024.2393948 (PMC11340745; doi:10.1080/15592294.2024.2393948)

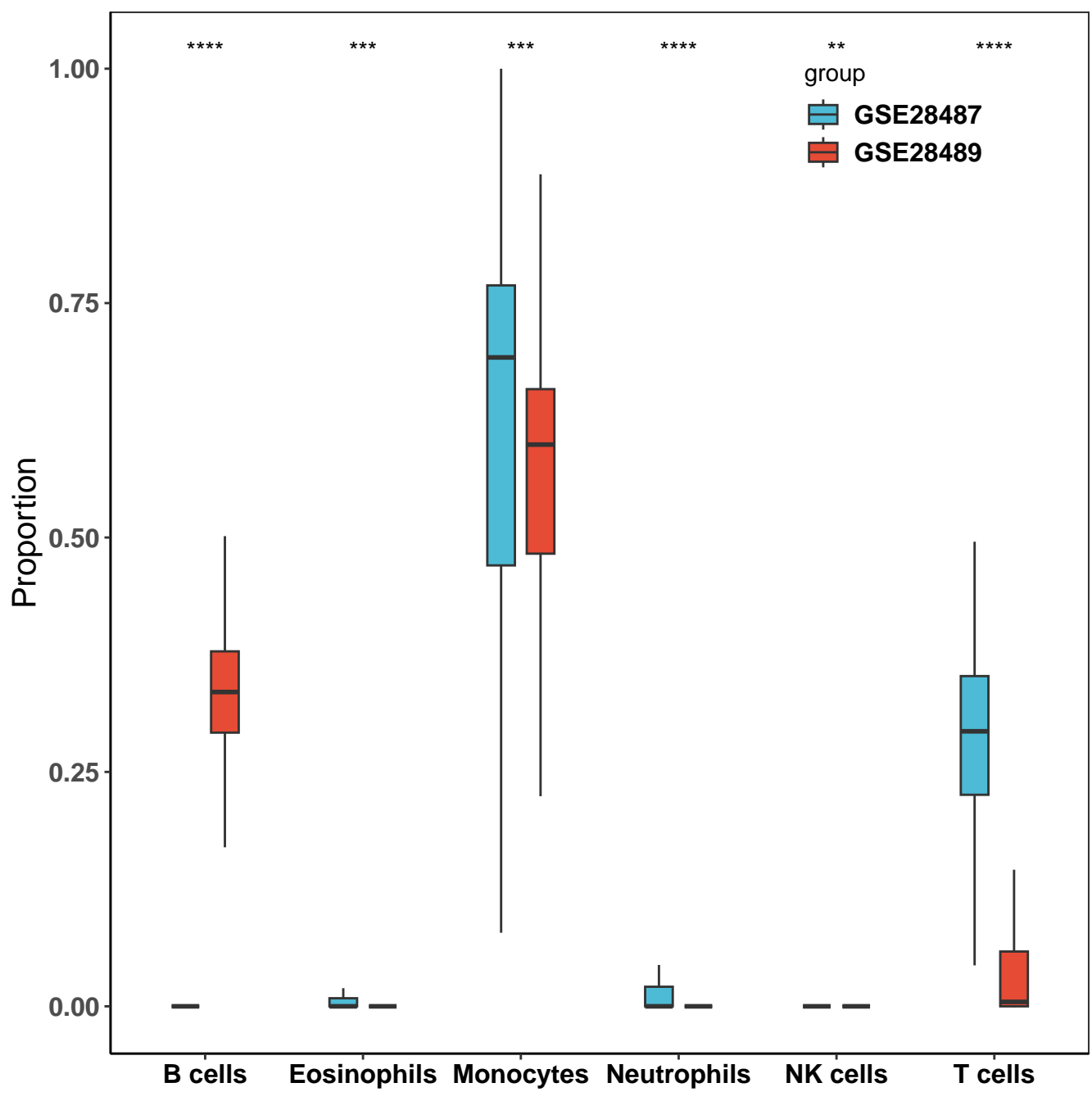

Supplement: Figure S1_CellProp boxplot.pdf [file KEPI_A_2393948_SM2160.pdf]
